# Induction of Immune Mediators in Glioma and Prostate Cancer Cells by Non-Lethal Photodynamic Therapy

**DOI:** 10.1371/journal.pone.0021834

**Published:** 2011-06-30

**Authors:** Robert Kammerer, Alexander Buchner, Patrick Palluch, Thomas Pongratz, Konstantin Oboukhovskij, Wolfgang Beyer, Ann Johansson, Herbert Stepp, Reinhold Baumgartner, Wolfgang Zimmermann

**Affiliations:** 1 Institute of Immunology, Friedrich Loeffler Institute, Tübingen, Germany; 2 Department of Urology, University Hospital of Munich, Munich, Germany; 3 Tumor Immunology Laboratory, LIFE Center, University Hospital of Munich, Munich, Germany; 4 Laser Research Laboratory, LIFE Center, University Hospital of Munich, Munich, Germany; City of Hope National Medical Center and Beckman Research Institute, United States of America

## Abstract

**Background:**

Photodynamic therapy (PDT) uses the combination of photosensitizing drugs and harmless light to cause selective damage to tumor cells. PDT is therefore an option for focal therapy of localized disease or for otherwise unresectable tumors. In addition, there is increasing evidence that PDT can induce systemic anti-tumor immunity, supporting control of tumor cells, which were not eliminated by the primary treatment. However, the effect of non-lethal PDT on the behavior and malignant potential of tumor cells surviving PDT is molecularly not well defined.

**Methodology/Principal Findings:**

Here we have evaluated changes in the transcriptome of human glioblastoma (U87, U373) and human (PC-3, DU145) and murine prostate cancer cells (TRAMP-C1, TRAMP-C2) after non-lethal PDT *in vitro* and *in vivo* using oligonucleotide microarray analyses. We found that the overall response was similar between the different cell lines and photosensitizers both *in vitro* and *in vivo*. The most prominently upregulated genes encoded proteins that belong to pathways activated by cellular stress or are involved in cell cycle arrest. This response was similar to the rescue response of tumor cells following high-dose PDT. In contrast, tumor cells dealing with non-lethal PDT were found to significantly upregulate a number of immune genes, which included the chemokine genes *CXCL2*, *CXCL3* and *IL8/CXCL8* as well as the genes for IL6 and its receptor IL6R, which can stimulate proinflammatory reactions, while IL6 and IL6R can also enhance tumor growth.

**Conclusions:**

Our results indicate that PDT can support anti-tumor immune responses and is, therefore, a rational therapy even if tumor cells cannot be completely eliminated by primary phototoxic mechanisms alone. However, non-lethal PDT can also stimulate tumor growth-promoting autocrine loops, as seen by the upregulation of IL6 and its receptor. Thus the efficacy of PDT to treat tumors may be improved by controlling unwanted and potentially deleterious growth-stimulatory pathways.

## Introduction

Photodynamic therapy (PDT) in oncology is based on the selective accumulation of a photosensitizer (PS) in cancer cells, followed by its activation by low-energy tissue-penetrating light. In the presence of oxygen, the excited PS produces reactive oxygen species (ROS) such as singlet oxygen, which are toxic for living cells [1].

Various PS exist which all have their advantages and their limitations. 5-Amino-levulinic acid (5-ALA) is a natural precursor for heme in mammalian cells. Cells metabolize 5-ALA to heme, producing protoporphyrin IX (PpIX) as an intermediate product in their mitochondria. Because conversion of PpIX to heme is a rate limiting step, PpIX accumulates in cancer cells as a result of preferential uptake and retention of 5-ALA. PpIX localizes intracellularly to mitochondria and to the cytoplasm [2]. Photofrin®, on the other hand, is an exogenous photosensitizer which also accumulates in cancer cells but is located at various cellular membranes [3]. In general, activation of PS which target mitochondria lead to cancer cell apoptosis, while PS that associate with cell membranes predominantly induce cell necrosis [2].

Originally, the goal of PDT in oncology was to completely eliminate localized tumors. However, the clinical application of PDT in the treatment of cancer has now begun to change. Recently, treatment regimes have been applied which seek to elicit vascular-targeting or anti-tumor immune effects [4]–[7]. This indicates that PDT could also be a rational treatment option for non-superficial tumors such as prostate cancer and glioblastoma.

Prostate cancer is the third leading cause of cancer related deaths for North American men [8]. The primary treatment of prostate cancer includes radical prostatectomy, androgen-deprivation therapy, radiotherapy and transperineal brachytherapy. All these treatments have a number of adverse effects which have a considerable impact on the patients [9]. Due to the dramatic improvements in early prostate cancer diagnosis focal treatments, such as cryotherapy, high-intensity focused ultrasound and PDT are emerging as new therapeutic options [10], [11]. The major drawback of focal therapy is the uncertainty of complete tumor cell eradication, especially since little is known about the response of tumor cells that escape from focal therapy. An unwanted scenario is that these tumor cells gain increased malignancy or start to secret factors that support proliferation or infiltration of residual cancer cells in a paracrine fashion.

Glioblastoma multiforme (GBM) is the most common and most aggressive type of primary brain tumors in humans. The median survival time of GBM patients is 14.6 months [12]. One of the characteristic features of glioblastomas is their diffuse infiltrative nature [13]. Recently it was observed that fluorescence-guided resection and repetitive PDT can significantly prolong median survival in patients with GBM [14], [15]. Furthermore, long-term survival of GBM patients after PpIX-based PDT was reported from other clinical studies [16], [17]. Despite these promising observations various issues have to be addressed to optimize PDT as a therapeutic option for GBM. For example, the response of tumor cells that survive PDT due to their advanced infiltration into the normal brain tissue is not well defined, but could be a target for an adjuvant therapy.

We have previously reported how the transcriptome of the prostate cancer cell line PC-3 responds to 5-ALA-based PDT [18]. Surprisingly PC-3 cells upregulated not only stress and DNA repair genes but also genes that code for proteins which are involved in the regulation of immune responses including certain chemokines and cytokines. Therefore, we wanted to know whether or not this observation holds true for other PS, for other prostate cancer cell lines, for tumor cells of another tissue origin and for tumors *in vivo*. To this end we comparatively analyzed the genome-wide transcriptional changes after low-level, non-lethal PDT in two human prostate and glioblastoma cell lines each, compared the transcriptome of the human prostate cancer line PC-3 after 5-ALA- and photofrin-based PDT and analyzed the transcriptional changes of murine prostate cancer cells subcutaneously transplanted into albino C57BL/6 mice upon PDT. We observed, independent of the tissue origin and the type of sensitizer, a marked transcriptional stimulation of genes coding for proinflammatory cytokines, which stimulate and attract predominantly myeloid leukocytes and, at the same time, activation of genes encoding proteins involved in cell cycle arrest. Taken together, the unavoidable incomplete destruction of tumor tissue by PDT under clinical settings might even support anti-tumor immune responses.

## Results

### Sensitization and irradiation conditions for non-lethal *in vitro* PDT

**Figure 1:**
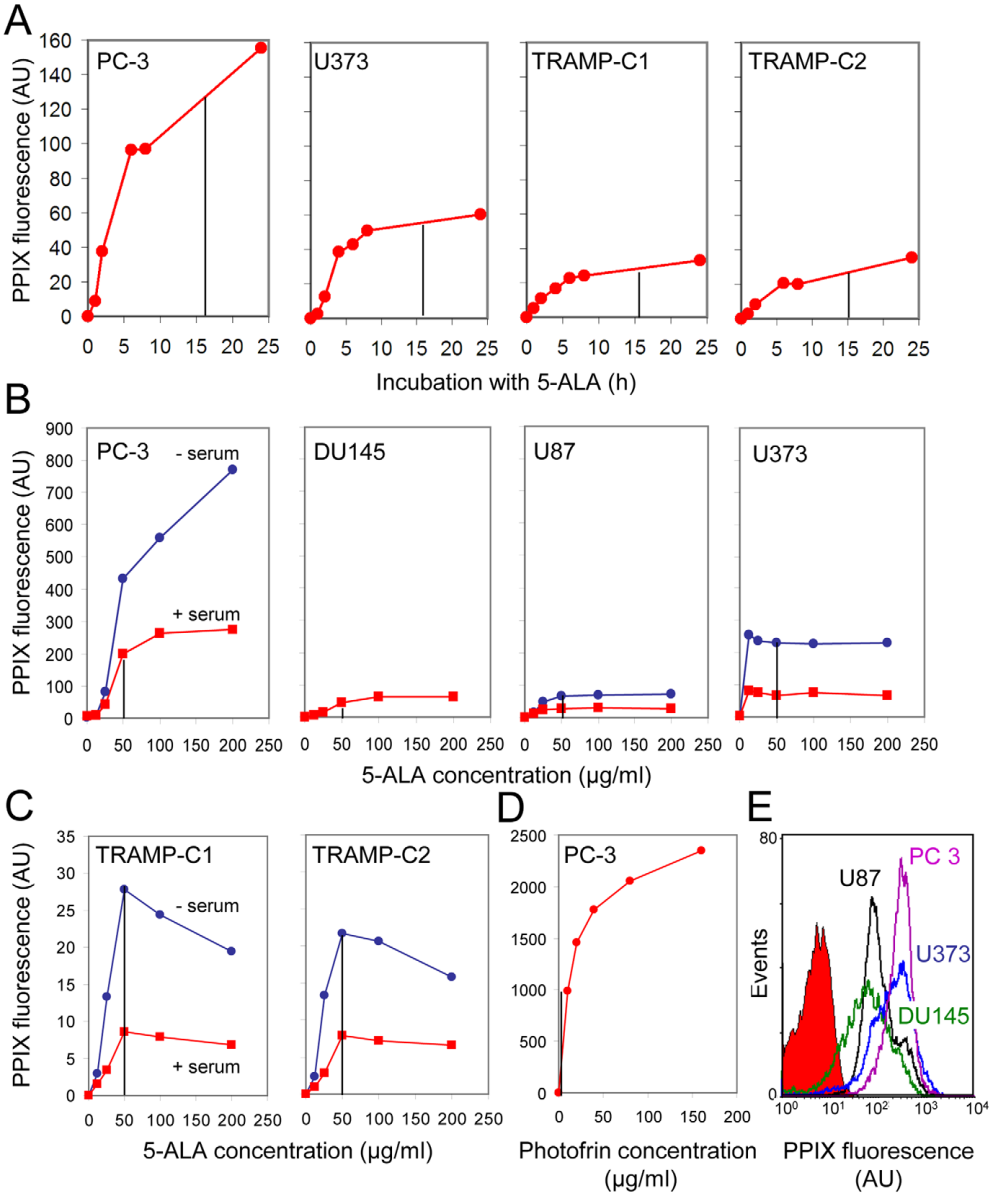
Kinetics and 5-ALA concentration and serum dependency of PpIX accumulation in glioma and prostate cancer cell lines. Tumor cells were incubated with 50 µg/ml (A, E) or with the indicated concentrations of 5-ALA (B, C) or photofrin (D) in the presence (PC-3, U373; A, D, E and red lines in B, C) or absence of 5% serum (TRAMP-C1, TRAMP-C2; A and blue lines in B, C). Unless otherwise indicated, the PpIX content of the cells was quantified after 16 h using flow cytometry in fluorescence channel 3 (median values ± standard deviation). Representative histograms for 5-ALA-treated human tumor cell lines are shown in E. Untreated cells were used as control; one representative control sample without 5-ALA incubation is shown (red filled-in curve). The photosensitizers showed saturable accumulation depending on incubation time and concentration. Serum in the culture medium strongly reduced ALA-based PpIX formation. The conditions for sensitizer loading used for the transcriptome analyses are indicated by vertical dotted lines. AU, arbitrary units.

In order to be able to reproducibly load tumor cells with photosensitizer we determined the kinetics and 5-ALA concentration dependence of PpIX formation in the tumor cell lines used. We selected two human prostate (PC-3, DU145) and two glioblastoma cell lines (U87, U373) as well as murine prostate carcinoma cell lines TRAMP-C1 and TRAMP-C2 which are derived from tumors of the transgenic mouse line “transgenic model of prostate cancer” (TRAMP) [19], [20]. The accumulation of photosensitizer was quantified by flow cytometry (Figure 1E). We noted large cell line-specific differences in the levels of PpIX formation after incubation with the PpIX precursor 5-ALA (Figure 1A-C, E). This could be due to differences in levels of the ATP-binding cassette transporter ABCG2 which is known to be responsible for export of PpIX from cells [21]. Indeed, the presence of ABCG2 mRNA in the different cell lines correlated with low levels of PpIX after incubation with 5-ALA with the highest amounts of ABCG2 mRNA being observed for TRAMP-C1 and TRAMP-C2 cells which exhibited the lowest levels of PpIX (Figure S1; Figure 1). In addition, the accumulation of PpIX was diminished 2–3 fold when the incubation with 5-ALA was performed in the presence of serum in the media (Figure 1B, C). This was probably due to binding of PpIX to serum albumin once transported out of the cell, thus shifting the PpIX steady state levels [22], [23]. Based on these data, a 16 h incubation period with 50 µg/ml 5-ALA in the presence or absence of 5% serum was chosen for the human prostate (PC-3, DU145) and the human glioblastoma and murine prostate cancer cell lines (U87, U373; TRAMP-C1, TRAMP-C2), respectively. A rather low but efficiently sensitizing photofrin concentration (5 µg/ml) was selected to avoid possible cytotoxicity in the absence of light (Figure 1D).

**Figure 2:**
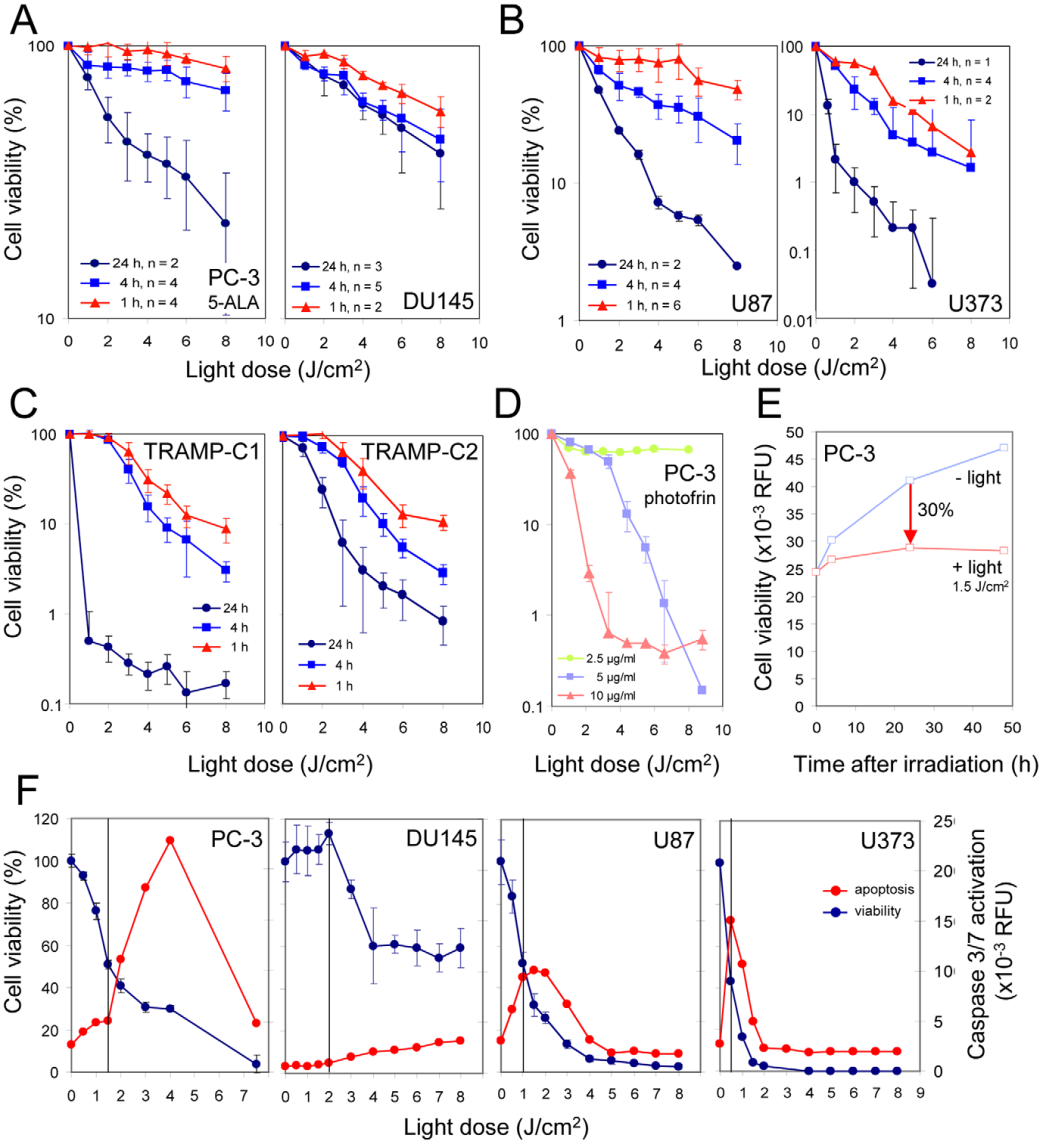
Irradiation conditions for PpIX- and photofrin-based non-lethal PDT. Human (A, B, D-F) or murine tumor cells (C) were sensitized by incubation with 50 µg/ml of 5-ALA (DU145: 100 µg/ml) or with the indicated concentrations of photofrin for 16 h in the presence (A, D, E; F, PC-3, DU145) or absence of 5% serum (B, C; F, U87, U373). Cells were irradiated with laser light (635 nm) delivering the indicated light doses. After the indicated times, cell viability and caspase3/7 activation were determined and displayed either as % of control for each time point (A–D, F) or in relative fluorescence units (RFU; E, F). Note the different propensity of prostate and glioma cells to suffer from apoptosis at low-dose PDT.

Next, light doses for irradiation of sensitized cells were defined which would allow cell survival for a period of 24 h to 48 h during which damaged cells could activate genes. We found that PDT conditions which caused a reduction of activity in the cell viability assay by 30% 24 h after PDT in comparison to a non-irradiated cell culture rather resulted in growth arrest with little or no loss of cells within a period of 48 h (Figure 2E). To determine the light dose inducing a 30% reduction of cell viability by measuring mitochondrial activity, cells were sensitized as described above and irradiated with increasing doses of laser light. Loss of cell viability increased with time after irradiation and light dose and differed between cell lines. For example, a 4-fold higher light dose was needed to induce a 30% reduction of cell viability 24 h after PDT for DU145 cells when compared to U373 or TRAMP-C1 cells (Figure 2A-D; Table S1). This differential sensitivity of the various cell lines did not correlate with their capability to accumulate PpIX in the presence of 5-ALA. Interestingly, the light doses leading to the same 30% loss of cell viability induced a different level of apoptosis in the analyzed human cell lines as measured by the activation of caspase 3 and caspase 7. Whereas no or a marginal level of apoptosis was observed in the prostate cancer cell lines DU145 and PC-3, respectively, maximal apoptosis was observed in the glioblastoma cell lines under the same conditions (Figure 2F).

### The transcription of a subset of chemokine and cytokine genes is highly upregulated in tumor cells after non-lethal *in vitro* PDT

**Figure 3:**
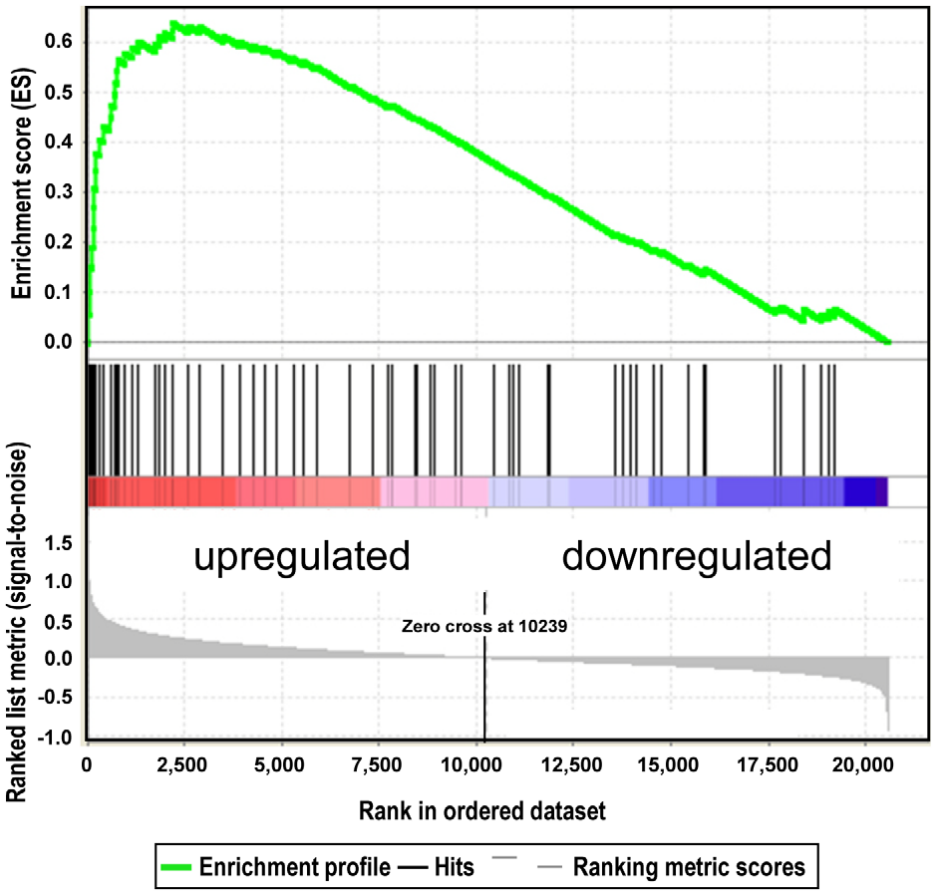
Non-lethal ALA-based PDT stimulates expression of neutrophil activation pathways in tumor cells. Gene set enrichment analysis (GSEA) revealed activation of a set of genes 24 h after non-lethal PDT of all 4 prostate and glioblastoma cell lines (data are shown for PC-3) which is also found to be characteristic for neutrophil activation during wound healing [55]. Upregulation is illustrated by the concentration of the vertical black lines (that represent gene set member positions within the ranked gene list) at the left side of the gene list (“zero cross”; see “waterfall plot” at the bottom of the graph). This distribution leads to a high and significant enrichment score (maximum deviation of the green line from zero; P<0.001 and false discovery rate (FDR) <0.001).

To determine the genes which are deregulated 4 h and 24 h after non-lethal PDT we performed transcriptome analysis of irradiated and non-irradiated human and murine tumor cells sensitized by incubation with 5-ALA (conditions are summarized in Table S1). A large fraction of probe sets/genes upregulated after PDT was shared between cell lines even between cell lines derived from tumors of various tissues of origin (e.g., ∼30–60% 24 h after PDT; Table S2, Table S3). Pathway analyses using the Gene Set Enrichment Analysis (GSEA) program revealed that 24 h after irradiation, out of a total of 3479 gene sets, 208 gene sets were significantly upregulated in glioblastoma cells, 156 gene sets in prostate cancer, and 508 gene sets in the combined samples (p<0.01) (Table S4). The sets of genes most prominently upregulated in all cell lines belong to pathways activated by cellular stress, including processes initiated by damage through ultraviolet light, hypoxia and ionizing radiation as well as gene sets, the encoded proteins that impede proliferation (“negative regulation of cell cycle”, “cell cycle arrest” and “apoptosis”; Table 1). Most significantly, a number of immune pathways were transcriptionally activated by non-lethal PDT, such pathways include “proinflammatory genes”, “interferon-β pathway” and “neutrophil activation in wound healing” (Table 1; Figure 3). The Gene Ontology gene sets most significantly downregulated in all analyzed cell lines were found to be associated with mitochondrial pathways (3 out of 5), which is in accordance with the mitochondria being the site of PpIX production and primary damage by ROS (Table S4).

**Table 1:** Selected gene sets (pathways) significantly activated in all cell lines 24 h after non-lethal PDT.

Curated gene sets	Gene Ontology gene sets
UV light response (24)	(Negative) regulation of cell cycle (4)
Ionizing radiation response (3)	Cell cycle arrest (2)
Hypoxia (5)	Apoptosis (4)
Viral infection response (14)	Immune system process
Interferon regulatory factor 4 targets (4)	Immune system development
Interferon-beta pathway	Myeloid cell differentiation
B lymphocyte development	B cell activation
Upregulated in lymphoid stem cells	
Proinflammatory genes	
Neutrophil activation in wound healing	

The expression of 374 gene sets from a total number of 2483 curated gene sets and 129 from a total number of 996 Gene Ontology gene sets were found to be significantly upregulated by GSEA analysis (P<0.01). Numbers in parentheses indicate the number of different upregulated curated gene sets or Gene Ontology gene sets within this subset.

**Figure 4:**
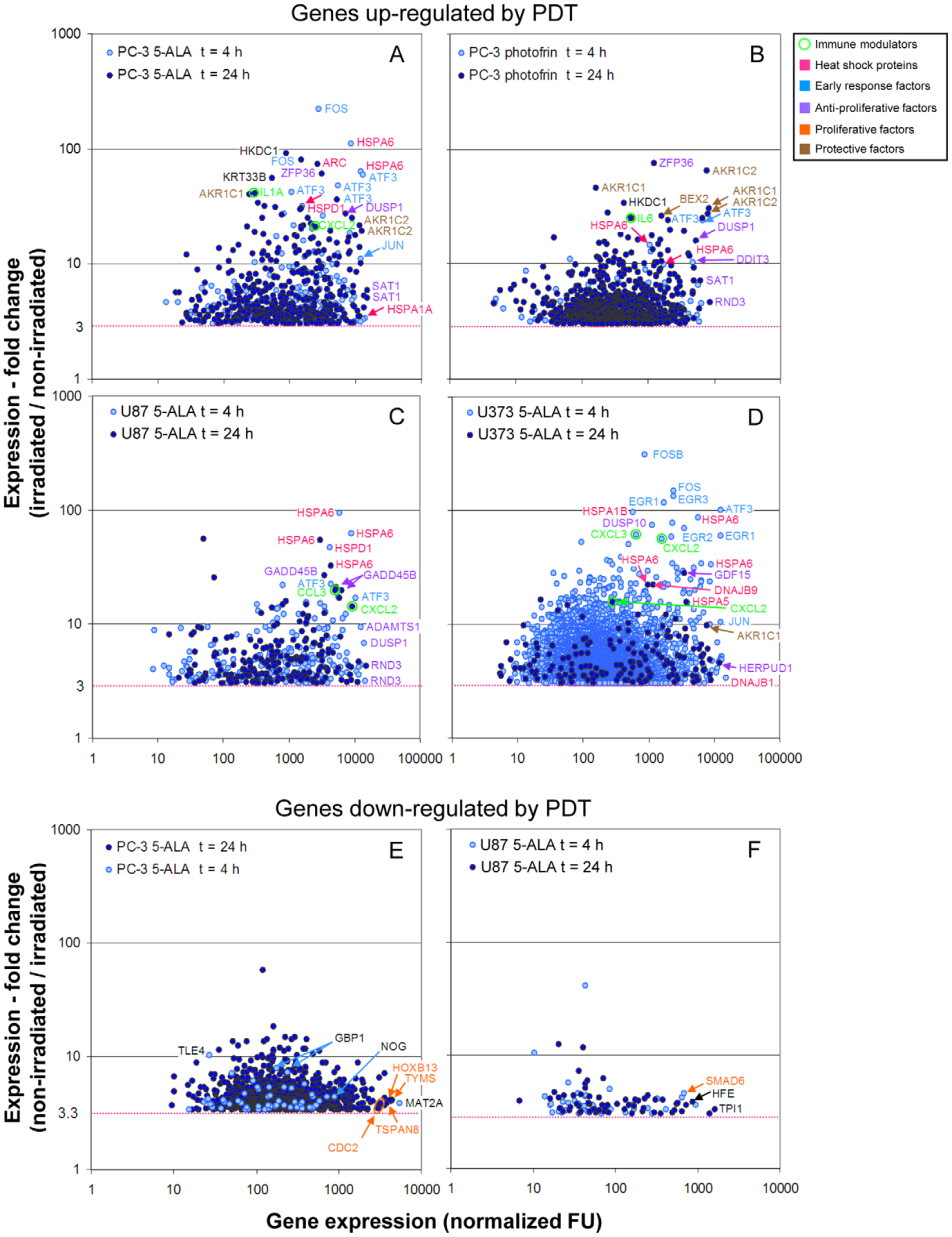
Genes transcriptionally deregulated in glioma and prostate cancer cell lines after non-lethal PDT. Tumor cells were subjected to non-lethal PDT after sensitization with 5-ALA (A, C–F) or photofrin (B). Total RNA was isolated 4 and 24 h after PDT and analyzed by hybridization to oligonucleotide microarrays. After normalization, fold change of gene expression between corresponding irradiated and non-irridiated samples was calculated and plotted against the expression level after PDT. Only genes (“probe sets”) are shown which were up- and down-regulated ≥3-fold and for which a ”present call" was registered for all irradiated and non-irradiated samples, respectively. The most strongly up-or down-regulated and most highly expressed genes in the samples are identified by gene symbols. Multiple depiction of gene symbols result from the presence of multiple probe sets for individual genes. Genes encoding immune modulatory proteins are additionally marked with small circles. Note that the latter genes are among the most strongly upregulated genes. A color code was used to discriminate groups of genes encoding functionally related proteins (see boxed Figure legend). For all samples mean expression values from duplicates were used. FU, fluorescence units.

Four h after non-lethal PDT, transcripts of “early response genes” were among the most strongly induced and most highly expressed genes in both human and murine tumor cells (Figure 4, Table S2, Table S3, Figure S2A–C). This group of genes encodes transcription factors like FBJ murine osteosarcoma viral oncogene homolog (FOS), JUN, activating transcription factor 3 (ATF3), early growth response 1 (EGR1) and DNA-damage-inducible transcript 3 (DDIT3). Upregulation of these genes in turn leads to transcription of typical stress genes like the heat shock protein (HSP) genes and can initiate G1 arrest and apoptosis (DDIT3) [24], [25]. Indeed, inducible members of the HSP gene family, namely heat shock 70 kDa protein 6 (*HSPA6*) followed in rank by *HSPA1A* and *HSPA1B* were the most dominantly induced genes 4 h after PDT in the human tumor cell lines (Figure 4A–D). In the murine TRAMP-C1 and TRAMP-C2 cells transcripts of the orthologs of the latter two members were prevalent 4 h after PDT (Figure S2B, C). The dominance of transcripts of early response transcription factor and HSP genes was lost 24 h after PDT, which was accompanied by increased activity of two major groups of genes involved in opposing cellular processes. One strongly stimulated and at high level expressed group of genes encodes proteins which exhibit anti-proliferative, pro-apoptotic and anti-invasive functions inducible by genotoxic stress like ROS (growth arrest and DNA-damage-inducible beta (GADD45B), dual-specific phosphatase 1 (DUSP1), ADAM metallopeptidase with thrombospondin type 1 motif 1 (ADAMTS1), homocysteine-inducible, endoplasmic reticulum stress-inducible ubiquitin-like domain member 1 (HERPUD1) [26], zinc finger protein 36 (ZFP36); growth differentiation factor 15/NSAI-activated gene 1 (GDF15/NAG-1 [27], spermidine/spermine N1-acetyltransferase 1 (SAT-1) [28]). The second group of genes codes for proteins which are known or supposed to enhance cell survival after cellular stress by inhibiting apoptosis and cell cycle arrest (*brain expressed X-linked 2* (*BEX2*) [25]) or by supporting detoxification of harmful compounds generated by ROS (*aldo-keto reductase 1C1*/*1C2* (*AKR1C1*/*AKR1C2*)) (Figure 4A–D).

**Figure 5:**
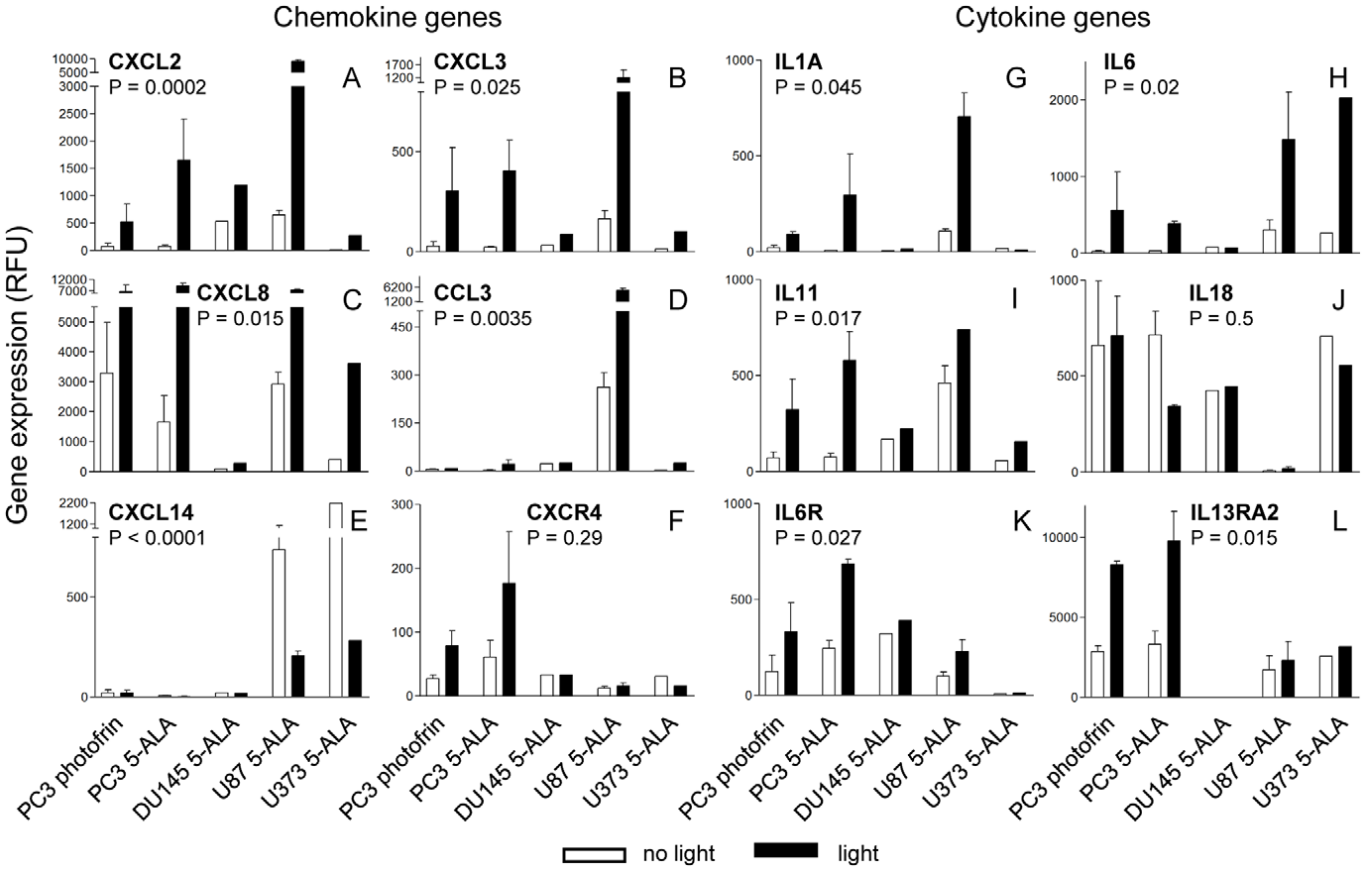
Transcription of genes encoding inflammatory interleukins and chemokines is enhanced after non-lethal PDT in tumor cell lines. Tumor cells were subjected to non-lethal PDT after sensitization with 5-ALA or photofrin and RNA was analyzed after 24 h recovery from PDT by hybridization to oligonucleotide microarrays as described in Legend to Figure 4. Mean expression values in relative fluorescence units (RFU) and their deviations for expressed interleukin and chemokine and interleukin and chemokine receptor genes are shown. For the *CXCL8* and *CXCL14* genes the 202859_x_at and 222484_s_at probe sets were used, respectively. For PC-3 and U87 samples mean expression values from duplicates, for DU145 and U373 samples single measurements were used. Significance levels (P) were calculated using Two-way ANOVA.

**Figure 6:**
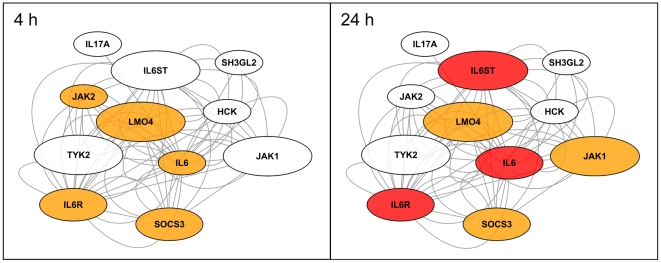
Transcriptional changes observed for genes encoding IL6-interacting proteins in PC-3 cells after PpIX-mediated PDT. The changes of gene expression 4 h and 24 h after PDT measured by oligonucleotide microarray analyses are depicted as fold change (fluorescence of irradiated/non-irradiated sample). The magnitude of fold change is indicated by different colors (<1.2-fold, white; ≥1.2-fold, <2-fold, orange; ≥2-fold, red). The strongest upregulation of expression was observed for the *IL6* gene (12.3-fold) which could part of an autostimulatory loop with the also upregulated IL6 receptor subunits (IL6R and IL6ST). The size of the ovals indicates the absolute expression level after PDT (<100 FU, small symbol; ≥100, <1000, medium symbol; ≥1000, large symbol). The lines between the ovals symbolize various types of interaction between the proteins or genes.

Interestingly, among the genes highly upregulated by the tumor cells after PDT were a number of immune response genes, notably chemokine and cytokine genes as well as chemokine and cytokine receptor genes (Figure 4, Figure 5). Expression of a substantial fraction of these genes was significantly upregulated 24 h after PDT often in both prostate and glioblastoma cell lines (P<0.05; two-way-ANOVA test). Among the most consistently upregulated genes were the chemokine genes *CXCL2*, *CXCL3* and *interleukin-8* (*IL8*)/*CXCL8* as well as the cytokine gene *IL6* (Figure 5A–C, H). Upregulation of the *CXCL2*, *CXCL3* genes was observed already 4 h after PDT in most cell lines (data not shown). Expression analysis of the network of gene products interacting with IL6 revealed a possible autostimulatory loop in PC-3 cells involving IL6 and its receptor subunits IL6R/IL6RA and IL6ST/IL6RB (Figure 6). Of note, expression of *CCAAT/enhancer binding protein β* (*CEBPB*) which encodes a transcription factor known to be involved in regulation of expression of *IL6*, was 3-fold and 4.2-fold induced 4 h and 24 h after PDT, respectively (not shown). Furthermore, genes encoding both negative and positive regulators of IL6 signaling namely suppressor of cytokine signaling 3 (SOCS3) and Janus kinase 1 (JAK1)/JAK2, respectively, were upregulated. For the *IL6* gene we have previously shown that transcriptional upregulation also translates into elevated secretion of the encoded protein by PC-3 cells after 5-ALA-based PDT [18]. No statistically significant activation was observed for some chemokine and cytokine genes (e.g., *CXCL1* (P = 0.25), *CCL26* (P = 0.24; data not shown); *IL18*, Figure 5J); *CXCL14* was found to be significantly downregulated (Figure 5E). To validate the expression data obtained by microarray oligonucleotide experiments we analyzed the mRNA levels of a selected set of genes (IL6, CXCL8 and CXCL14) in total RNA from PDT-treated and control cells 24 h after irradiation. We found a good agreement between the two sets of expression values determined by the two different quantitation methods (Figure S3, Figure 5). This is reflected by coefficients of determination R^2^ close to 1.0 (0.947±0.072) and a similar statistically significant up- (IL6, CXCL8) and down-regulation (CXCL14) as found using the oligonucleotide microarray data set.

However, there is also a fraction of genes (∼25%) which was preferentially upregulated (≥4-fold) in PC-3 or U87 cell and thus might exhibit tissue-of-origin-specific stimulation. The most differentially stimulated genes are either already comparatively highly expressed in the unresponsive cell line (e.g., *EGR1*, *IL11*, *AKR1C1-3* in U87; CD55 in PC-3) or probably do not belong to the repertoire of expressible genes in the respective cell line (*nuclear receptor subfamily 4 group A member 1* (*NR4A1*), *tumor necrosis factor-α-induced protein 6* (*TNFAIP6*), *dehydrogenase/reductase (SDR family) member 9* (*DHRS9*), *cyclin-dependent kinase inhibitor 1A* (*Kip2*) in U87) (Table S5).

In comparison, only few genes/probe sets were downregulated >3-fold after PDT (Figure 4E, F and data not shown). The most strongly expressed genes that were downregulated often encoded cell growth and survival promoting proteins.

### ROS-inducible genes are more efficiently upregulated by 5-ALA-based PDT than by photofrin-mediated PDT

Interestingly, although endogenously produced PpIX and the synthetic oligomeric porphyrin photofrin are thought to act in different cellular compartments [3], a similar set of genes was upregulated both at 4 h and 24 h after non-lethal PDT in PC-3 cells (Figure 4A, B). However, despite a similar level of inhibition of cell viability 4 h after non-lethal PDT (5-ALA 15±3%; photofrin 17.5±0.5%; Table 1) 14% (28/204) of the genes/probe sets, which were more than 3-fold upregulated after 5-ALA-based PDT, exhibited a ≥4-fold higher transcriptional activation in comparison to that observed after photofrin-mediated PDT (Table S6). The preferentially upregulated genes that were expressed at a high level comprised, among other genes, early response genes (*FOS*), HSP genes (*HSPD1, HSPA6*), cell survival genes (histone cluster 1 genes), as well as immune response genes (*IL1A*, *CCL26*). In contrast, no gene strongly expressed after PDT (>1000 RFU) was selectively stimulated by photofrin-based PDT (Table S6).

### Transcriptional upregulation of proinflammatory genes in TRAMP-C2 tumors after non-lethal PDT

**Figure 7:**
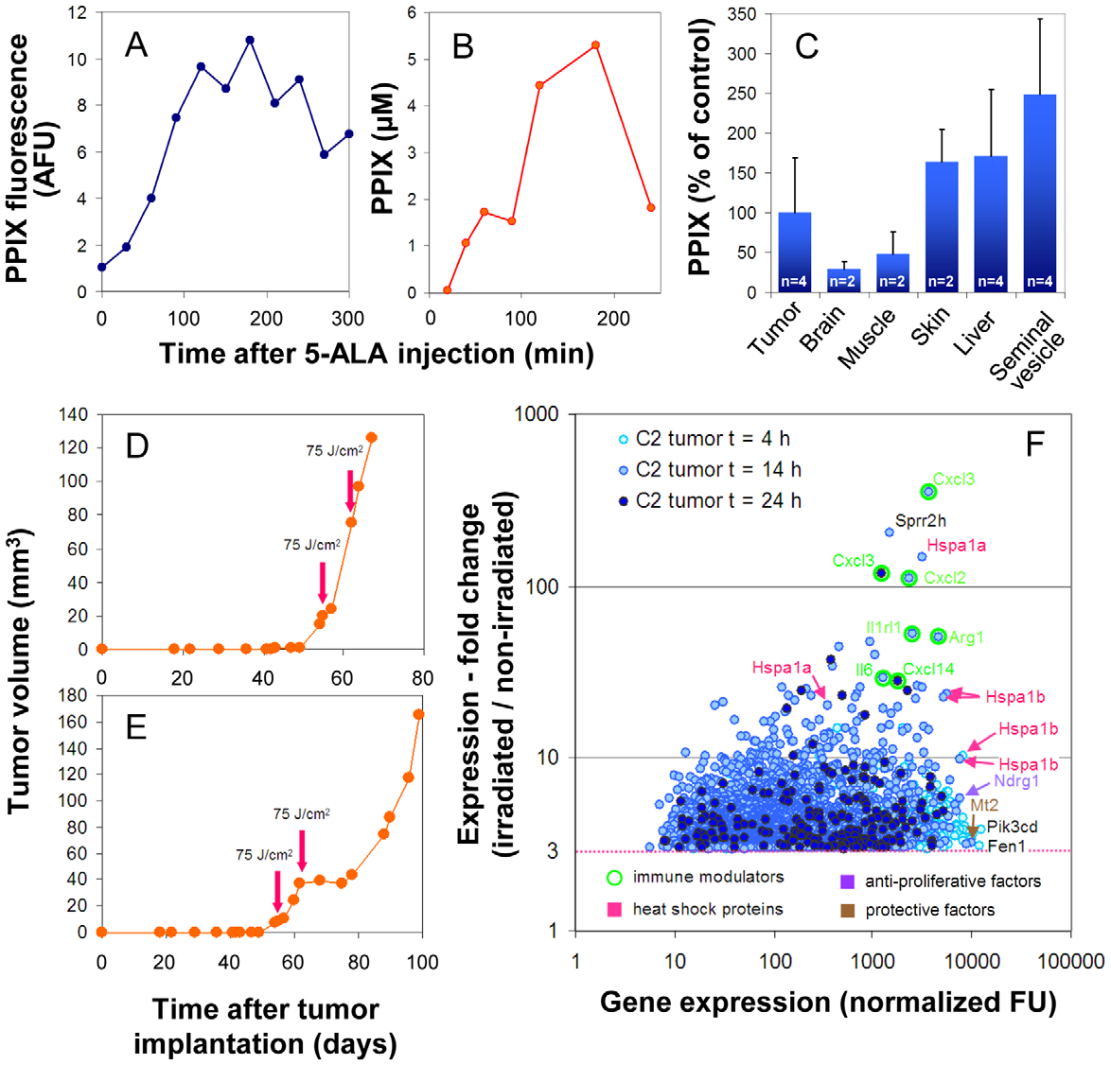
Transcription of genes encoding inflammatory interleukins and chemokines is enhanced after non-lethal PDT in murine C2 tumors. The kinetics of PpIX accumulation in mouse tissues after i.p. injection of 5-ALA was determined either by direct measurement of PpIX fluorescence in the skin covering the tumor (A) or by fluorescence spectroscopical determination of PpIX in tumor extracts from a single mouse each which was sacrificed at the indicated time (B). PpIX accumulation in different tissues relative to TRAMP-C2 tumors 3.5 h after 5-ALA injection is shown in (C). In (A), a typical kinetic of PpIX accumulation in skin is shown. Maximal accumulation of PpIX in skin and tumor tissue was observed ∼180 min after 5-ALA injection. Tumors were subjected to PDT (75 J/cm^2^) after sensitization with 5-ALA for 180 min. Note, that 5-ALA-mediated PDT after maximal sensitization had only a marginal (D) or transient inhibitory effect on tumor growth (E) even after duplicate application one week apart. For transcriptome analysis, sensitized tumors from a single mouse each were treated or not with laser light (635 nm; 75 J/cm^2^), excised 4, 14 or 24 h after irradiation. RNA was isolated and analyzed by hybridization to oligonucleotide microarrays. After normalization, fold change of gene expression between irradiated and non-irridiated samples was calculated and plotted against the expression level after PDT (F). Only genes (“probe sets”) are shown which were upregulated ≥3-fold and for which a „present call“ was registered in the irradiated sample. The most strongly upregulated and most highly expressed genes in the samples are identified by gene symbols. Multiple depiction of gene symbols result from the presence of multiple probe sets for individual genes. Genes encoding immune modulatory proteins are additionally marked with small circles. AFU; arbitrary fluorescence units; n, number of mice tested.

Since the expression of chemokine and cytokine genes were consistently upregulated in cell lines after 5-ALA-based PDT *in vitro*, we examined whether 5-ALA-based PDT could also induce the expression of proinflammatory genes in tumors, which could support anti-tumor immune reactions. We used as a tumor model murine TRAMP-C2 prostate tumor cells grown subcutaneously in albino C57BL/6 mice, which allowed transdermal irradiation of the tumor with visible light. First we optimized sensitization conditions by measuring the accumulation of PpIX in the skin over the tumor after i.p. injection of 5-ALA as an approximation for PpIX formation in tumor tissue. In most mice, maximal PpIX levels in the skin were reached after 2–3 h (Figure 7A). A similar kinetic of PpIX accumulation was observed by spectrophotometric determination of PpIX in tumor extracts (Figure 7B).

Compared to TRAMP-C2 tumors, relative high levels of PpIX were found in extracts of liver, seminal vesicle and skin (Figure 7C). This might be due to the strong expression of the PPIX efflux transporter gene *Abcg2* in C2 tumors and the C2 tumor cell line (Figure S1) and could explain the dose-limiting side effects seen in the mice after PDT. Consequently, the light doses which could be applied led to an inefficient control of tumor growth (Figure 7D, E). Transcriptome analyses using RNA isolated from irradiated and non-irradiated whole tumors 14 h and 24 h after PDT revealed that the fraction of highly transcriptionally activated genes after non-lethal PDT comprised mostly genes encoding proinflammatory factors (Figure 7F; Figure S2). As observed for PDT-treated tumor cell lines, *Cxcl2*, *Cxcl3* and *Il6* were among the most strongly upregulated genes, although we cannot exclude that these cytokines were expressed by stromal cells in the tumor microenvironment or tumor-associated immune cells. Interestingly, *Sprr2h* (*small proline-rich protein 2*) a gene known to be regulated by IL6/STAT3 signaling [29] is the second most strongly induced gene 14 h after PDT (Figure 7F). Four h after PDT, *Hsp1a* and *Hsp1b* belonged to the group of most strongly activated and most highly expressed genes (Figure 7F).

## Discussion

Various reasons exist why non-lethal PDT is often applied unintendedly to tumor cells *in vivo*. One of these reasons is that cells within a tumor are often heterogeneous and can differ in their ability to accumulate PS as well as in their susceptibility to oxidative stress. Indeed, we observed a striking variability of PpIX accumulation between the different tumor cell lines used. This is not restricted to tumor cells *in vitro*, but was also observed in tumor cells *in vivo* [30], [31]. In the present study we identified genes that are upregulated in cancer cells upon non-lethal PDT *in vitro* and compared them to genes which were affected within tumor tissues *in vivo*. Importantly, we found that cancer cells independent of their tissue origin (prostate and brain) by themselves upregulate immune-related genes as a response to PDT-induced cell stress. This indicates that oxidative stress induces similar responses in different cell types, all aimed to limit the sensitivity of the cells to ROS, to repair the inflicted cell defects and to coordinate immune responses intended to clear the irreversibly damaged tissue from the organism. This finding has important implications for the use of PDT as a treatment option for both GBM and prostate cancer: (I) our results indicate that the overall response of different tumors to non-lethal PDT is similar between tumors originating from different tissue types; (II) tumor cells respond to non-lethal PDT by upregulating the expression of immune function genes which may support proinflammatory anti-tumoral immune responses; (III) PDT could be especially suitable for immunogenic tumors where the benefit from tumor immunity for the patient is larger than the possible disadvantage caused by a higher concentration of tumor growth-promoting factors associated with inflammation.

Early transcriptional changes elicited by non-lethal PDT mirrored the changes previously observed using various PS and PDT high-dose settings [32]–[36]. Early response genes, stress genes/heat shock protein genes, growth arrest/DNA damage response/proapoptotic/antiproliferative genes but also cell survival/antiapoptotic/proangiogenetic genes were already upregulated 4 h after non-lethal PDT (Table S2). Of note after 24 h a few unique genes were upregulated not seen upon high-dose PDT, due to the fact that most if not all tumor cells did not survive high-dose PDT for 24 h. Among these genes were immune response genes, including *IL1A*, *IL6*, *CXCL2*, *CXCL3* and *CXCL8*/*IL8*, some of which appeared to be preferentially upregulated by PpIX, rather than by photofrin-mediated PDT (Table S6). All these genes have effects on the recruitment and/or activation of granulocytes. It is conceivable that due the different location of the PS within cell compartments and the short half-life of ROS generated by PS activation resulting in localized damage of different cell compartments similar but not identical gene activation programs are initiated. This could also lead to differential activation of cellular damage repair programs associated with increased resistance to PDT. Resistance to photofrin-based PDT in U87 cells was recently reported to be caused by stimulation of expression of *ALKBH2* (alkylated DNA repair protein alkB homolog 2 gene) [37]. However, 5-ALA-based PDT with U87 cells did not activate this repair process in our experiments (data not shown). Therefore, PpIX PDT might combine two advantages by inducing immune mediators more efficiently and at the same time being less prone to activate repair processes. Buytaert and colleagues recently investigated the transcriptional changes in bladder cancer cells upon hypericin-mediated low-dose PDT and observed upregulation of the *granulocyte-macrophage colony-stimulating factor* (*CSF2*) and *TLR2* genes besides *IL8* [38]. Whether this difference is due to the use of hypericin as PS, the different time point of measurements after PDT or the different cell lines analyzed is currently unknown. In addition, the clear upregulation of the *matrix metalloproteinase-1* (*MMP1*), *MMP10* and *MMP13* genes reported by these authors was not seen in our experiments. This result is unexpected, since the upregulated transcription factors and stress response genes were similar in both studies. It could well be that different cell types, in addition to commonly upregulated genes, have an individual repertoire of genes which they can express following PDT. Indeed, we observed a cell type-specific upregulation of a number of genes such as *AKR1C1-3*, *CD55*, *IL11*, *TNFAIP6* and others in our investigation. Whatever the reasons for the different findings, it reminds us to be cautious assessing the potential impact of the transcriptional changes after PDT on tumor cell behavior. Indeed, there are other reports suggesting that photofrin-mediated PDT using subtherapeutic light doses can promote growth and infiltration of GBM [39], [40]. However, there is increasing evidence from clinical data that suggests that GBM patients can benefit from PpIX-based PDT by significantly longer median survival [14], [15]. More importantly, it was reported that there are long-term survivors within the cohort of patients that were treated with PDT [16], [17], [41]. Indeed, when we analyzed changes in the transcriptome of TRAMP-C2 tumors *in vivo*, we noticed a robust upregulation of immune response genes, including *Il6*, *Cxcl2* and *Cxcl3* within the tumors (Figure 7F). Whether this upregulation of expression takes place in tumor cells or cells of the tumor microenvironment was not determined. However, since the same immune mediators were upregulated by prostate and glioblastoma cell lines *in vitro*, this suggests that tumor cells cooperate in attracting granulocytes to the region of PDT. Indeed, granulocyte infiltration was observed in tumors treated with non-lethal PDT by us (data not shown) and others [42], [43]. It has been suggested that induction of immune related genes in tumor cells upon PDT is due to NF-κB activation [44]. Among the hundreds of NF-κB target genes, some may also favor tumor cell growth and dissemination. Especially, the *prostaglandin endoperoxide synthase 2* gene (*PTGS2/COX-2*) and matrix metalloproteinase genes are important in this context. They were, however, not found to be upregulated more than three fold in our experimental system. On the other hand, some tumors have acquired the ability to use the IL6/IL6 receptor (IL6R)/signal transducer and activator of transcription 3 (STAT3)/hypoxia-inducible factor 1α (HIF1α) signaling cascade to be able to proliferate faster [45], [46]. We have observed that expression of *IL6* as well as *IL6R* is significantly upregulated in both prostate and glioblastoma cancer cells by PDT. Furthermore, IL6ST, the shared receptor of many cytokines, is expressed in these cells and the *JAK1* gene is also upregulated 24 h after PDT. Hence the elevated level of IL6 in the tumor microenvironment, besides exerting an immune stimulatory effect, may also support tumor growth and thereby limit the benefit gained by anti-tumor immune responses [47]. Interestingly, in both prostate cancer and GBM the IL6-STAT3 axis is thought to play a tumor-promoting role [45], [48]. Disruption of this novel autocrine loop (IL6/STAT3/HIF1α) with appropriate drugs may enhance the therapeutic effect of PDT [49]. Indeed, clinical trials are underway where the impact of IL6-targeting antibodies on prostate cancer and other tumors is being evaluated (www.clinicaltrials.gov).

Taken together, our results suggest that non-lethal PDT supports anti-tumor immune responses and, therefore, PDT seems to be a suitable therapy, especially for immunogenic tumors, even if a complete eradication of tumor cells by phototoxicity alone is not guaranteed. Further elucidation of tumor-specific and PS-selective variations of the transcriptional response after PDT is desirable to minimize the risk of harmful adverse effects through induced autocrine tumor-promoting loops.

## Materials and Methods

### Ethics Statement

Animal studies were approved by the local regulatory agency (Regierung von Oberbayern, Munich, Germany; approval ID 55.2-1-54-2531-109-06).

### Cell lines and cell culture

Human prostate carcinoma cell lines PC-3 and DU145 (DMSZ, German Collection of Microorganisms and Cell Cultures, Braunschweig, Germany), and the human glioblastoma cell lines U87 MG (Institut für angewandte Zellkultur, Dr. Toni Lindl GmbH, München, Germany) and U373 MG (ATCC) as well as murine prostate carcinoma cell lines TRAMP-C1 and TRAMP-C2 (LGC Standards GmbH, Wesel, Germany) were cultured in RPMI-1640 medium supplemented with 5% fetal calf serum (FCS “Gold”; PAA Laboratories, Coelbe, Germany), 2 mM L-glutamine, 100 U/ml penicillin, 100 µg/ml streptomycin, non-essential amino acids and 1 mM sodium pyruvate (GIBCO/Invitrogen, Karlsruhe, Germany) at 37°C with 5% CO_2_ in a humidified atmosphere. Absence of mycoplasma contamination war determined using the VenorGeM Kit (Minerva Biolabs, Berlin, Germany).

### Optimization of photosensitizer generation and loading

Tumor cells (6×10^4^ per well) were plated in 24-well plates (Nunc GmbH, Wiesbaden, Germany) and grown over night in the presence of 5% FCS. Cells were washed with FCS free medium and incubated with varying 5-ALA concentrations (Medac GmbH, Hamburg, Germany) in the presence or absence of 5% serum for the indicated times. 5-ALA stock solutions (1 mg/ml) were freshly prepared in serum free medium. Photofrin (Axam Pharma International BV, Quebec, Canada) sensitization of cells was measured in the presence of 5% FCS. Photofrin stock solutions (3.75 mg/ml in 0.9% NaCl solution) were stored at 4°C in the dark. Cells were removed by treatment with 200 µl of 0.05% trypsin/0.53 mM EDTA (Invitrogen, Paisley, UK), spun down at 500 x g for 5 min after addition of 1 ml of 5% FCS-containing medium and resuspended in 200 µl of Hank's balanced salt solution. PpIX and photofrin fluorescence was induced by a 635 nm red diode laser and measured in the FL3 photomultiplier tube (670 nm long pass filter) of a FACSCalibur flow cytometer (BD Biosciences, Erembodegem, Belgium) and its median was used after subtraction of the median of the fluorescence of unlabeled cells as a measure of the PpIX or photofrin content in the tumor cells. To exclude underestimation of the PPIX concentrations by FACS measurements due to quenching at relatively high intracellular PPIX levels control experiments using quantitation of PPIX by fluorescence spectrometry after extraction from cells and dilution were performed. Both methods proved to be equivalent (Figure S4). In all experiments with sensitized cells, care was taken to minimize exposure of the cells to ambient light.

### *In vitro* PDT treatment

For determination of the light dose suitable for induction of a ∼20% and ∼30% loss of cell viability 4 h and 24 h after PDT, respectively, 1×10^4^ cells per well were plated in flat bottom 96-well plates (Nunc GmbH, Wiesbaden, Germany) in 4–6 replicates per data point and grown for 6–8 h in medium with 5% FCS. Sensitization of the cells with 2.5–10 µg/ml photofrin or 10–200 µg/ml 5-ALA was performed for 16 h in 100 µl of fresh medium with (PC3, DU145) or without (U87, U373) 5% FCS. The medium was replaced with 100 µl of RPMI-1640 without phenol red (Invitrogen/Gibco) containing supplements and 5% serum as listed above. Specific light doses were delivered to the cells by irradiation (six wells at the time) with laser light (600 µm fiber with attached microlens, Biolitec AG, Jena, Germany and a 635 nm Ceralas diode laser, CeramOptec GmbH, Bonn, Germany) at 100 mW/cm^2^ for varying times in a light tight box with a 37°C warm plate. Because of higher light sensitivity of photofrin-sensitized cells, irradiation with different light doses was performed on separate microtiter plates to exclude effects from scattered light on cells in neighboring wells. For the isolation of RNA from PDT-treated cells, tumor cells (1×10^6^) were grown in 4 ml medium in 6 cm diameter cell culture dishes (Nunc), sensitized as above with 50 µg/ml 5-ALA (DU145: 100 µg/ml) or 5 µg/ml photofrin and irradiated at room temperature. The following light doses (J/cm^2^) were used: PC-3: 1.5 and 3; DU145: 2; U87: 1; U373: 0.5; TRAMP-C1: 0.5; TRAMP-C2: 0.8. Separate identically treated cell cultures were used for cell viability determination (see below). After PDT, cells were incubated in the same medium at 37°C for the indicated times.

### PDT of murine tumors

Tumors were induced by subcutaneous injection of 1-3×10^6^ TRAMP-C2 tumor cells in 50 µl of phosphate-buffered saline (PBS) in the lumbar region of syngeneic male albino C57BL/6 mice (C57BL/6-Tyr^c-2J^). The mice were kindly provided by H. Schrewe (Max-Planck Institute for Molecular Genetics, Berlin, Germany). The size of tumors was determined twice a week with a caliper and the approximate volume was calculated by using the formula: length x width^2^/2. Tumors-bearing mice (tumor volume: 100–300 mm^3^) were injected with 15 mg 5-ALA/ml PBS (500 mg/kg) under isoflurane anesthesia. During sensitization and a period of 3 days following PDT mice were kept in the dark. The 5-ALA solution was freshly prepared before each experiment, neutralized using 10 N NaOH and stored in the dark on ice. PpIX accumulation in the skin covering the tumor was determined by measuring the fluorescence intensity at 635 nm using an irradiation/detection system consisting of a laser diode with an emission wavelength of 405 nm, a bifurcated fiber (Light Guide Optics, Rheinbach, Germany), a filter (GG435, Schott AG, Mainz, Germany) and a fiber spectrophotometer (S2000 Mikropack, Ocean Optics, Ostfildern, Germany). PpIX formation in tumor and in other tissues as well as for comparison in PC-3 cells was also determined by fluorescence spectroscopy after extraction from shock-frozen tissues or cells protected from light and stored at −80°C as described recently [30]. For the calculation of the molar PpIX concentration a tissue density of 1 g/cm^3^ was assumed. Three to 4 h after injection of 5-ALA, tumor-bearing mice were narcotized with xylazine/ketamine, shaven at their back and irradiated after application of a tight fitting aperture plate cut from black cardboard. The whole tumor area was irradiated perpendicular through the intact skin using the same laser light source as described for *in vitro* PDT, however, applying 75 or 100 J/cm^2^ at 200 mW/cm^2^. After treatment, tumors were either excised, shock-frozen and stored at −80°C or their growth was followed as described above.

### Measurement of cell viability, apoptosis and survival

After PDT, cell viability was quantified using the CellTiter-Blue™ Cell Viability Assay (Promega, Mannheim, Germany), apoptosis was measured using the Apo-ONE® Homogeneous Caspase-3/7 Assay (Promega) as recommended by the manufacturer. In brief, after the indicated times 200 µl of CellTiter-Blue™ or 100 µl Apo-ONE® solution per ml were directly added to the cell culture medium without phenol red, mixed gently and after 1 h, samples in 96-well plates were measured directly or 1 ml of the cell culture supernatant was removed and stored in the dark at 4°C (up to 24 h). Fluorescence was quantified in 96-well cell culture plates (100 µl per well) in a FLUOstar OPTIMA microplate reader (BMG LABTECH, Jena, Germany) using an excitation/emission filter of 560/590 and 485/520 nm for the Cell Titer Blue™ and Apo-ONE® assay, respectively. The background fluorescence values were obtained by addition of CellTiter-Blue™ or Apo-ONE® solution to medium without cells and incubation for 1 h.

### RNA labeling and oligonucleotide microarray hybridization

PDT-treated cells or cells identically treated except for irradiation were detached with 1 ml of trypsin/EDTA, washed as above and stored at −80°C. Total RNA was isolated from the cell pellets or tumors using the RNeasy Mini Kit (Qiagen, Hilden, Germany). The RNA yield was quantified photometrically and the integrity was determined by capillary electrophoresis (Agilent 2100 Bioanalyzer and RNA 6000 Pico Kit; Agilent Technologies Deutschland, Böblingen, Germany). The RNA integrity number (RIN) provided by the Agilent system allows a quantitative estimate of the RNA quality. The RIN number (10 represents intact RNA) for all samples was >9.5 [50]. RNA amplification and biotin labeling was performed by reverse transcription of 1–5 µg of total RNA with an oligo-dT-T7 promoter primer and linear *in vitro* transcription using a kit from Affymetrix (One Cycle Target Labeling Kit) according to the manufacturer's instructions. Hybridization of biotin-labeled RNA to Affymetrix Gene Chip U133 Plus 2.0 or Mouse Genome 430 2.0 oligonucleotide arrays was done for 16 h at 37°C in a hybridization oven (Affymetrix). Then, the arrays were washed according to the standard protocol and stained by addition of streptavidin-phycoerythrin using the Fluidics Station 450 (Affymetrix). Laser scanning of the arrays was performed using the GeneChip® Scanner 3000 7G (Affymetrix). Raw hybridization data (probe cell intensity) were stored as CEL files. The CEL files and normalized microarray data from this study are MIAME compliant and are available at the ArrayExpress Archive (accession numbers: E-MEXP-3016 [murine cells, 8 arrays], E-MEXP-3017 [murine tumors, 7 arrays] and E-MEXP-3020 [human cells, 32 arrays]). The Expression Console™ 1.1 Software (Affymetrix) was used for initial data quality control and calculation of the detection call (transcript “absent” or “present”).

### Microarray data analysis

CEL file data were used to determine a model-based expression index (MBEI) with the approach of Li and Wong that is implemented in the dChip 2009 software (http://www.dchip.org; Dana-Farber Cancer Center and Harvard School of Public Health, Boston, MA, USA) [51]. This algorithm has shown good performance in processing of raw microarray data in a comparative study of seven methods [52]. The MBEI values were used for subsequent high-level analysis (e.g., hierarchical clustering). In addition, for the identification of pathways with significantly changed expression between sample groups Gene Set Enrichment Analysis (GSEA 2.0; Broad Institute, Harvard/MIT; software available from http://www.broadinstitute.org/gsea) was used [53]. The GSEA algorithm can identify subtle, but significant and biologically relevant expression changes of functionally coupled genes between two sample groups. GSEA uses a database with >6000 gene sets (MSigDB v3.0, molecular signature database, Broad Institute, Harvard/MIT). A gene set consists of genes that belong to the same pathway (e.g., signal transduction, metabolism) or that have other features in common (e.g., chromosomal localization). For this study, only curated gene sets (from published studies) and Gene Ontology terms with 15–500 genes were used, leaving 3479 gene sets (2483 curated and 996 from Gene Ontology) for analysis.

Brief description of the GSEA algorithm: First, all genes are ranked according to their signal-to-noise ratio [(µ1–µ2)/(σ1+σ2); µ, mean; σ, standard deviation] between the two phenotype groups (e.g., 24 h after irradiation without/with prior sensitization). Then, every gene set is tested for significant functional enrichment of its genes. The algorithm goes through the ranked gene list, and a running-sum statistic is increased when a gene is part of the gene set, and decreased if it is not. The magnitude of the increment depends on the correlation of the gene with the phenotype. The enrichment score is the maximum deviation from zero encountered in walking the list. The enrichment score is near 0 if the genes of a gene set are distributed equally over the ranked gene list. In case of functional enrichment, many genes are “concentrated” at one end of the ranked gene list because their expression correlates with one phenotype and the enrichment score is different from 0 (positive or negative). Permutation tests are performed to calculate a p value and the false discovery rate (FDR) for each gene set.

Visualization of gene expression changes in pathways was performed using the software Cytoscape 2.7.0 ([54]; software available at http://www.cytoscape.org) and interaction data sets from Pathway Commons (http://www.pathwaycommons.org, built and maintained by Memorial Sloan-Kettering Cancer Center, New York and the University of Toronto). Network nodes (genes) were color-coded according to their expression fold change between nonsensitzed and sensitized samples 4 h and 24 h after irradiation.

### Quantitative RT-PCR

One µg of total RNA was reverse transcribed using the Reverse Transcription System from Promega according to the manufacturer's protocol. PCR amplification was performed using 40 ng of cDNA, specificity-tested primers (LightCycler™ Primer Sets; Search LC, Heidelberg) and the LightCycler™ FastStart DNA MasterPLUS Sybr Green I kit (Roche, Penzberg, Germany). The relative abundance of transcripts was calculated in arbitrary units (AU) using the formula 2^−Cp^×10^10^ (AU) whereby Cp represents the crossing point where a fluorescence value of 1 is reached.

### Statistics

Significance levels (P) were calculated using Two-way ANOVA in the GraphPad Prism3 software package. Comparisons of samples exhibiting P values <0.05 were considered to be significantly different.

## Supporting Information

Figure S1 **ABCG2 mRNA levels in human and murine prostate tumor cell lines negatively correlate with PpIX accumulation.** The amount of ABCG2 mRNA was determined by oligonucleotide microarray analyses in human (PC-3) and murine prostate cancer cell lines (TRAMP-C1, TRAMP-C2) as well as in subcutaneously grown murine prostate tumors (TRAMP-C2). As a control, the expression of the house keeping gene encoding the TATA-box-binding protein (TBP) is show. Note the high levels of expression of the PpIX exporter ABCG2 in murine prostate cancer cells which inversely correlates with their ability to accumulate PpIX in the presence of 5-ALA (see Fig. 1A, C; Fig. 6B, C). Mean and (standard) deviations are shown. n = 2 for murine cells, n = 3 for PC-3.
(PPT)

Figure S2 **Genes transcriptionally upregulated in human glioma (A) and murine prostate cancer cells (B, C) and tumors after non-lethal PDT (D).** Tumor cells and tumors were subjected to non-lethal PDT after sensitization with 5-ALA. Tumors were irradiated with a light dose of 75 or 100 J/cm2. Total RNA was isolated 4 and 24 h after PDT and analyzed by hybridization to oligonucleotide microarrays. After normalization, fold change of gene expression between corresponding irradiated and non-irridiated samples was calculated and plotted against the expression level after PDT. Only genes (probe sets) are shown which were up- and down-regulated ≥3-fold and for which a „present call“ was registered for all irradiated and non-irradiated samples, respectively. The most strongly up- or down-regulated and most highly expressed genes in the samples are identified by gene symbols. Multiple depiction of gene symbols result from the presence of multiple probe sets for individual genes. Genes encoding immune modulatory proteins are additionally marked with small circles. A color code was used to discriminate groups of genes encoding functionally related proteins (see boxed Figure legend). FU, fluorescence units.
(PPT)

Figure S3 **Validation of expression of selected cytokine genes by quantitative RT-PCR.** Total RNA was isolated from glioblastoma and prostate cancer cells (DU145, n = 1; all other cell lines n = 2) 24 h after photofrin (PC-3) or 5-ALA-based PDT (conditions are summarized in Table S1). Control cells were also incubated with photofrin or 5-ALA, however, were not irradiated. The relative cDNA level of IL6 (A), CXCL8 (B) and CXCL14 (C) were determined by quantitative RT-PCR. The thus determined cDNA levels were correlated with the levels estimated from oligonucleotide microarray experiments shown in Fig. 5. A high degree of agreement between the two different quantitation methods was noted (coefficient of determination R2 = 0.947±0.072). Significance levels (P) were calculated using two-way ANOVA.
(PPT)

Figure S4 **Equivalence of PPIX quantitation in PC-3 cells by flow cytometry or extraction and photometric measurement.** 3×105 cells were incubated for 16 h with different 5-ALA concentrations in the presence of 10% FCS. PPIX content was either determined after extraction of the cell pellets with 100 µl the aqueous based solubilizer Solvable™ (PerkinElmer) and further 100-fold dilution with Solvable™ by fluorescence spectroscopy (A) or by flow cytometry (FL3 photomultiplier tube; 670 nm long pass filter) (B). The concentrations of the final dilutions were calculated from a standard curve obtained by dilution of purified PPIX in Solvable™. The median of the PPIX fluorescence of the strongest labeled cell fraction is indicated. Both measurements proved to be highly equivalent as demonstrated by a coefficient of determination (R2) close to 1 (C).
(PPT)

Table S1 **Non-lethal PDT conditions for transcriptome analysis of human and murine prostate and glioblastoma cell lines.**
(DOC)

Table S2 **Genes upregulated in both prostate cancer PC-3 and glioblastoma U87 cells 4 h and 24 h after non-lethal 5-ALA-based PDT.**
(XLS)

Table S3 **Genes upregulated in both glioblastoma U87 and U373 cells 24 h after non-lethal 5-ALA-based PDT.**
(XLS)

Table S4 **Significantly deregulated gene sets (pathways) in prostate and glioblastoma tumor cell lines 24 h after PDT.**
(DOC)

Table S5 **Genes preferentially upregulated in prostate cancer PC-3 cells or in glioblastoma U87 cells 24 h after non-lethal 5-ALA-based PDT.**
(XLS)

Table S6 **Genes preferentially upregulated 4 h and 24 h after non-lethal 5-ALA- versus photofrin-based PDT in PC-3 cells.**
(XLS)
