# Supplementary material for: Induction of Immune Mediators in Glioma and Prostate Cancer Cells by Non-Lethal Photodynamic Therapy
Source: PLoS One. 2011 Jun 30;6(6):e21834. doi: 10.1371/journal.pone.0021834 (PMC3128096; doi:10.1371/journal.pone.0021834)
Supplement: Figure S1 — ABCG2 mRNA levels in human and murine prostate tumor cell lines negatively correlate with PpIX accumulation. The amount of ABCG2 mRNA was determined by oligonucleotide microarray analyses in human (PC-3) and murine prostate cancer cell lines (TRAMP-C1, TRAMP-C2) as well as in subcutaneously grown murine prostate tumors (TRAMP-C2). As a control, the expression of the house keeping gene encoding the TATA-box-binding protein (TBP) is show. Note the high levels of expression of the PpIX exporter ABCG2 in murine prostate cancer cells which inversely correlates with their ability to accumulate PpIX in the presence of 5-ALA (see Fig. 1A, C; Fig. 6B, C). Mean and (standard) deviations are shown. n = 2 for murine cells, n = 3 for PC-3. (PPT) [file pone.0021834.s001.ppt]

## Slide 1
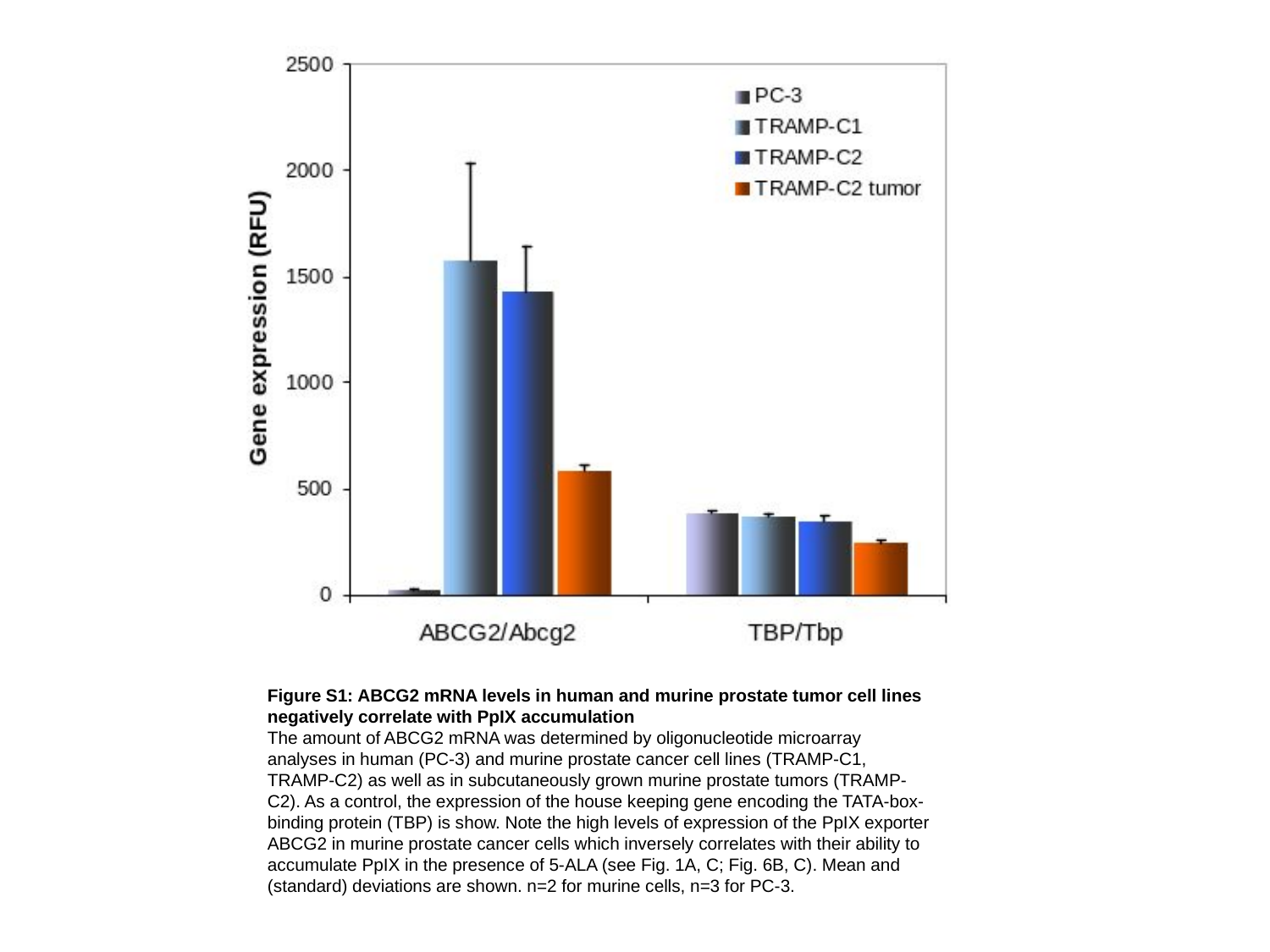

Figure S1: ABCG2 mRNA levels in human and murine prostate tumor cell lines negatively correlate with PpIX accumulation
The amount of ABCG2 mRNA was determined by oligonucleotide microarray analyses in human (PC-3) and murine prostate cancer cell lines (TRAMP-C1, TRAMP-C2) as well as in subcutaneously grown murine prostate tumors (TRAMP-C2). As a control, the expression of the house keeping gene encoding the TATA-box-binding protein (TBP) is show. Note the high levels of expression of the PpIX exporter ABCG2 in murine prostate cancer cells which inversely correlates with their ability to accumulate PpIX in the presence of 5‑ALA (see Fig. 1A, C; Fig. 6B, C). Mean and (standard) deviations are shown. n=2 for murine cells, n=3 for PC-3.
