# Supplementary material for: Induction of Immune Mediators in Glioma and Prostate Cancer Cells by Non-Lethal Photodynamic Therapy
Source: PLoS One. 2011 Jun 30;6(6):e21834. doi: 10.1371/journal.pone.0021834 (PMC3128096; doi:10.1371/journal.pone.0021834)
Supplement: Figure S2 — Genes transcriptionally upregulated in human glioma (A) and murine prostate cancer cells (B, C) and tumors after non-lethal PDT (D). Tumor cells and tumors were subjected to non-lethal PDT after sensitization with 5-ALA. Tumors were irradiated with a light dose of 75 or 100 J/cm2. Total RNA was isolated 4 and 24 h after PDT and analyzed by hybridization to oligonucleotide microarrays. After normalization, fold change of gene expression between corresponding irradiated and non-irridiated samples was calculated and plotted against the expression level after PDT. Only genes (probe sets) are shown which were up- and down-regulated ≥3-fold and for which a „present call“ was registered for all irradiated and non-irradiated samples, respectively. The most strongly up- or down-regulated and most highly expressed genes in the samples are identified by gene symbols. Multiple depiction of gene symbols result from the presence of multiple probe sets for individual genes. Genes encoding immune modulatory proteins are additionally marked with small circles. A color code was used to discriminate groups of genes encoding functionally related proteins (see boxed Figure legend). FU, fluorescence units. (PPT) [file pone.0021834.s002.ppt]

## Slide 1
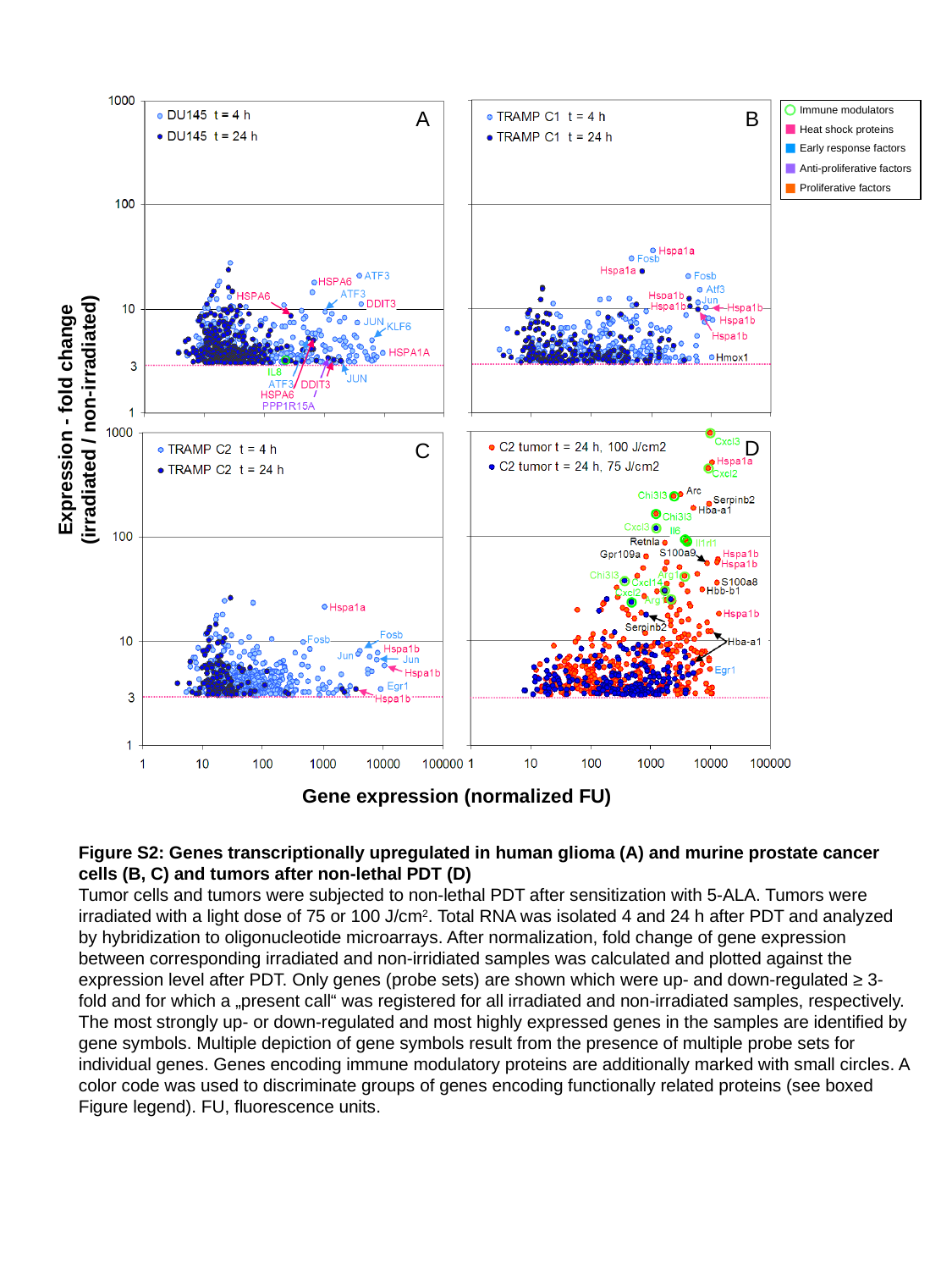

A
B
Immune modulators
Heat shock proteins
Early response factors
Anti-proliferative factors
Proliferative factors
Expression - fold change
(irradiated / non-irradiated)
D
C
Gene expression (normalized FU)
Figure S2: Genes transcriptionally upregulated in human glioma (A) and murine prostate cancer cells (B, C) and tumors after non-lethal PDT (D)
Tumor cells and tumors were subjected to non-lethal PDT after sensitization with 5-ALA. Tumors were irradiated with a light dose of 75 or 100 J/cm2. Total RNA was isolated 4 and 24 h after PDT and analyzed by hybridization to oligonucleotide microarrays. After normalization, fold change of gene expression between corresponding irradiated and non-irridiated samples was calculated and plotted against the expression level after PDT. Only genes (probe sets) are shown which were up- and down-regulated ≥ 3-fold and for which a „present call“ was registered for all irradiated and non-irradiated samples, respectively. The most strongly up- or down-regulated and most highly expressed genes in the samples are identified by gene symbols. Multiple depiction of gene symbols result from the presence of multiple probe sets for individual genes. Genes encoding immune modulatory proteins are additionally marked with small circles. A color code was used to discriminate groups of genes encoding functionally related proteins (see boxed Figure legend). FU, fluorescence units.
