# Supplementary material for: Induction of Immune Mediators in Glioma and Prostate Cancer Cells by Non-Lethal Photodynamic Therapy
Source: PLoS One. 2011 Jun 30;6(6):e21834. doi: 10.1371/journal.pone.0021834 (PMC3128096; doi:10.1371/journal.pone.0021834)
Supplement: Figure S3 — Validation of expression of selected cytokine genes by quantitative RT-PCR. Total RNA was isolated from glioblastoma and prostate cancer cells (DU145, n = 1; all other cell lines n = 2) 24 h after photofrin (PC-3) or 5-ALA-based PDT (conditions are summarized in Table S1). Control cells were also incubated with photofrin or 5-ALA, however, were not irradiated. The relative cDNA level of IL6 (A), CXCL8 (B) and CXCL14 (C) were determined by quantitative RT-PCR. The thus determined cDNA levels were correlated with the levels estimated from oligonucleotide microarray experiments shown in Fig. 5. A high degree of agreement between the two different quantitation methods was noted (coefficient of determination R2 = 0.947±0.072). Significance levels (P) were calculated using two-way ANOVA. (PPT) [file pone.0021834.s003.ppt]

## Slide 1
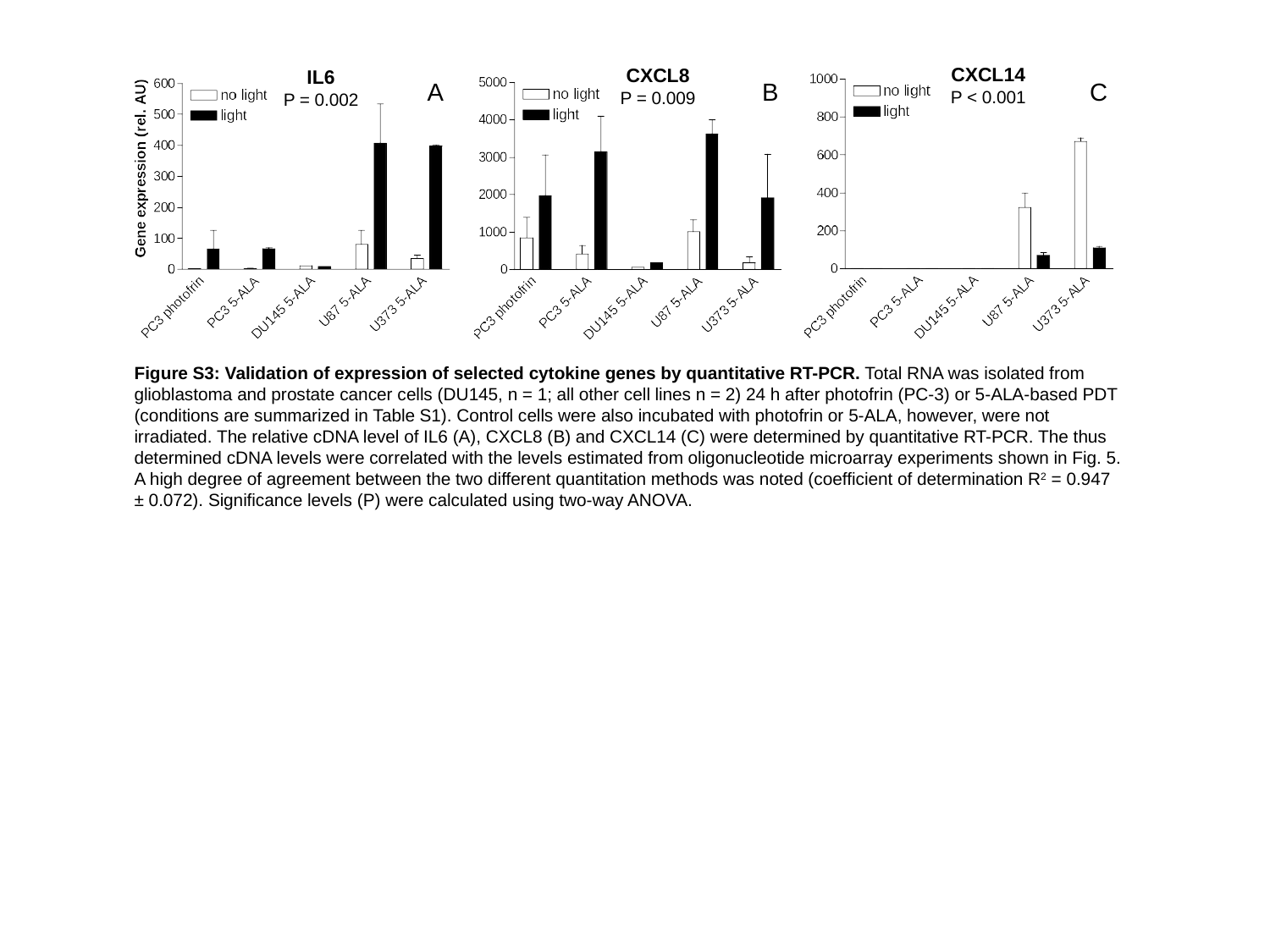

CXCL14
P < 0.001
CXCL8
P = 0.009
IL6
P = 0.002
A
B
C
Figure S3: Validation of expression of selected cytokine genes by quantitative RT-PCR. Total RNA was isolated from glioblastoma and prostate cancer cells (DU145, n = 1; all other cell lines n = 2) 24 h after photofrin (PC-3) or 5-ALA-based PDT (conditions are summarized in Table S1). Control cells were also incubated with photofrin or 5-ALA, however, were not irradiated. The relative cDNA level of IL6 (A), CXCL8 (B) and CXCL14 (C) were determined by quantitative RT-PCR. The thus determined cDNA levels were correlated with the levels estimated from oligonucleotide microarray experiments shown in Fig. 5. A high degree of agreement between the two different quantitation methods was noted (coefficient of determination R2 = 0.947 ± 0.072). Significance levels (P) were calculated using two-way ANOVA.
