# Supplementary material for: Induction of Immune Mediators in Glioma and Prostate Cancer Cells by Non-Lethal Photodynamic Therapy
Source: PLoS One. 2011 Jun 30;6(6):e21834. doi: 10.1371/journal.pone.0021834 (PMC3128096; doi:10.1371/journal.pone.0021834)
Supplement: Figure S4 — Equivalence of PPIX quantitation in PC-3 cells by flow cytometry or extraction and photometric measurement. 3×10^5^ cells were incubated for 16 h with different 5-ALA concentrations in the presence of 10% FCS. PPIX content was either determined after extraction of the cell pellets with 100 µl the aqueous based solubilizer Solvable™ (PerkinElmer) and further 100-fold dilution with Solvable™ by fluorescence spectroscopy (A) or by flow cytometry (FL3 photomultiplier tube; 670 nm long pass filter) (B). The concentrations of the final dilutions were calculated from a standard curve obtained by dilution of purified PPIX in Solvable™. The median of the PPIX fluorescence of the strongest labeled cell fraction is indicated. Both measurements proved to be highly equivalent as demonstrated by a coefficient of determination (R2) close to 1 (C). (PPT) [file pone.0021834.s004.ppt]

## Slide 1
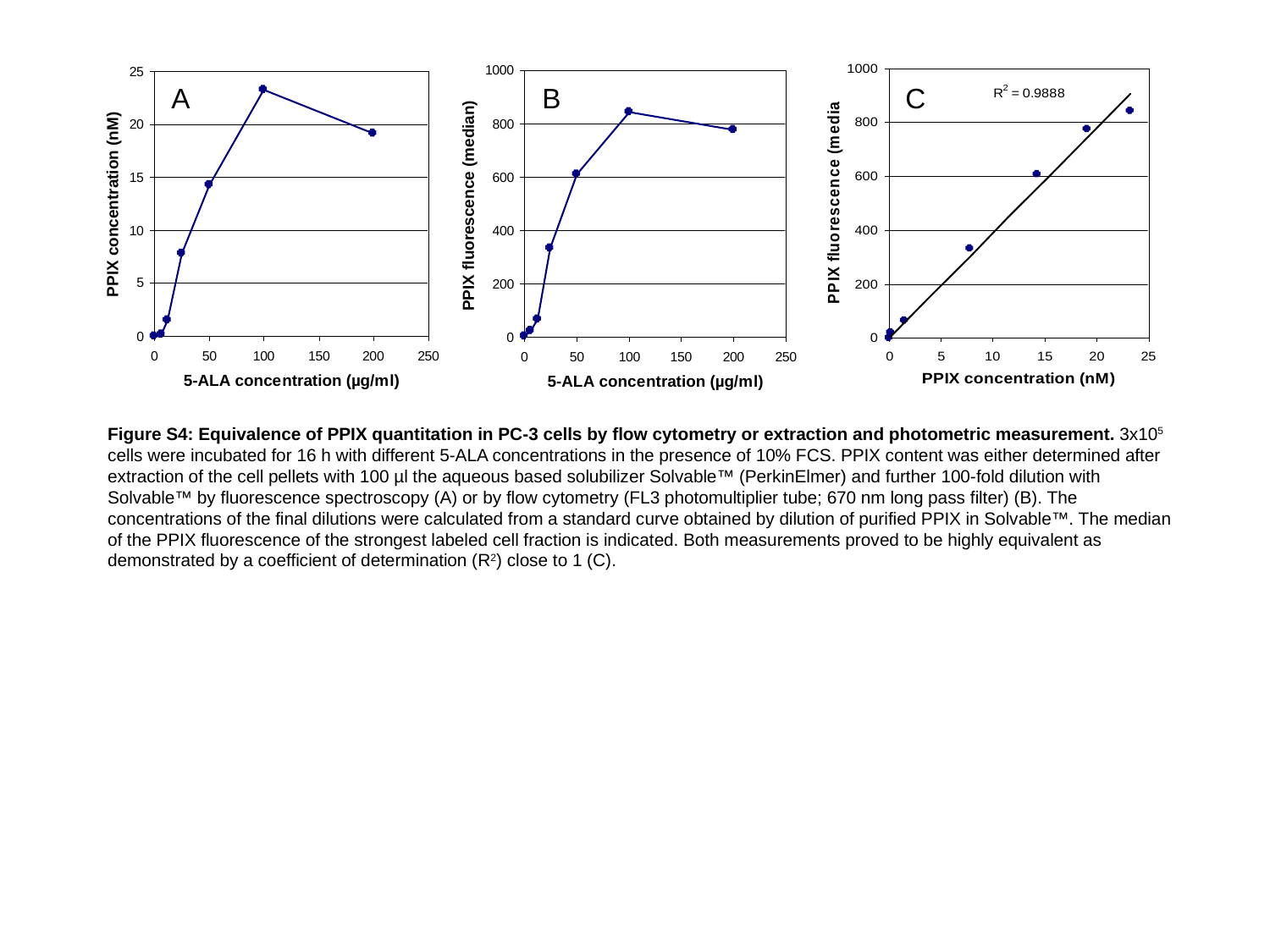

A
B
C
Figure S4: Equivalence of PPIX quantitation in PC-3 cells by flow cytometry or extraction and photometric measurement. 3x10^5^ cells were incubated for 16 h with different 5-ALA concentrations in the presence of 10% FCS. PPIX content was either determined after extraction of the cell pellets with 100 µl the aqueous based solubilizer Solvable™ (PerkinElmer) and further 100-fold dilution with Solvable™ by fluorescence spectroscopy (A) or by flow cytometry (FL3 photomultiplier tube; 670 nm long pass filter) (B). The concentrations of the final dilutions were calculated from a standard curve obtained by dilution of purified PPIX in Solvable™. The median of the PPIX fluorescence of the strongest labeled cell fraction is indicated. Both measurements proved to be highly equivalent as demonstrated by a coefficient of determination (R2) close to 1 (C).
