# Supplementary material for: Induction of Immune Mediators in Glioma and Prostate Cancer Cells by Non-Lethal Photodynamic Therapy
Source: PLoS One. 2011 Jun 30;6(6):e21834. doi: 10.1371/journal.pone.0021834 (PMC3128096; doi:10.1371/journal.pone.0021834)
Supplement: Table S1 — Non-lethal PDT conditions for transcriptome analysis of human and murine prostate and glioblastoma cell lines. (DOC) [file pone.0021834.s005.doc]

**Table S1:** **Non-lethal PDT conditions for transcriptome analysis of human and murine prostate and glioblastoma cell lines.**

| **Cell line** | **sensitizer** | **serum concentration** | **light dose** | **inhibition of viability after PDT** | |
| --- | --- | --- | --- | --- | --- |
|  |  |  |  | 4 h | 24 h |
| PC-3 ^1^ | 5-ALA | 5% | 1.5 J/cm² | 15.0 ± 3.0% | 33.9 ± 3.0% |
| PC-3 ^1^ | Photofrin® | 5% | 1 J/cm² | 17.5 ± 0.5% | 16.5 ± 5.5% |
| DU145 | 5-ALA | 5% | 2 J/cm² | 3.0% | 27.0% |
| U87 MG^1^ | 5-ALA | 0% | 1 J/cm² | 18.5 ± 1.5% | 26.5 ± 0.5% |
| U373 MG^1^ | 5-ALA | 0% | 0.5 J/cm² | 17.5 ± 0.5% | 29.5 ± 3.5% |
| TRAMP-C1 | 5-ALA | 0% | 0.5 J/cm² | 20.0% | 31.3% |
| TRAMP-C2 | 5-ALA | 0% | 0.8 J/cm² | 4.0% | 32.7% |

^1^ determined in duplicates
