# Supplementary material for: Induction of Immune Mediators in Glioma and Prostate Cancer Cells by Non-Lethal Photodynamic Therapy
Source: PLoS One. 2011 Jun 30;6(6):e21834. doi: 10.1371/journal.pone.0021834 (PMC3128096; doi:10.1371/journal.pone.0021834)
Supplement: Table S4 — Significantly deregulated gene sets (pathways) in prostate and glioblastoma tumor cell lines 24 h after PDT. (DOC) [file pone.0021834.s008.doc]

**Table S4:** **Significantly deregulated gene sets (pathways) in prostate and glioblastoma tumor cell lines 24 h after PDT.**

|  | **Number of upregulated / downregulated curated gene sets**  (total number: 2483) | | **Number of upregulated / downregulated Gene Ontology gene sets** (total number: 996) | |
| --- | --- | --- | --- | --- |
| Significance level | p < 0.01 | FDR < 0.05 | p < 0.01 | FDR < 0.05 |
| Glioblastoma cell lines | 177/85 | 109/0 | 31/23 | 0/0 |
| Prostate cancer cell lines | 138/139 | 57/185 | 18/37 | 0/51 |
| All cell lines | 374/27 | 330/6 | 129/5 | 54/0 |

GSEA was conducted with 1000 permutations per analysis run using 3479 predefined gene sets. FDR, false discovery rate.
